# National Cancer Institute and the 2023 -2025 Estimate – Cancer Incidence in Brazil

**DOI:** 10.1055/s-0043-1762925

**Published:** 2023-03-06

**Authors:** Walquíria Quida Salles Pereira Primo

**Affiliations:** 1 Universidade de Brasília, Brasília DF, Brazil

As part of the celebration of the National Cancer Awareness Day (November 27), the launch of the 2023 estimate of the incidence of cancer in Brazil was on November 23, 2022 at the headquarters of the National Cancer Institute (INCA) in Rio de Janeiro, including the 21 most frequent tumors in the country. The estimate is an important planning and management tool in oncology in Brazil and provides fundamental information for the definition of public policies. 1

During the three-year period (2023–2025), of the total of 704,000 new cases of cancer each year in the country, excluding non-melanoma skin cancer, an estimated 244,000 occur in women and 239,000 in men. Regarding regions, 70% of cases are expected for the South and Southeast regions. Breast cancer in women (South: 71.44/100 thousand; Southeast: 84.46/100 thousand), prostate cancer (South: 57.23/100 thousand; Southeast: 77.89/100 thousand) and colon and rectum (South: 26.46/100 thousand; Southeast: 28.75/100 thousand) are the three most frequent types in these two regions. 1 2

In the North and Northeast regions, prostate cancer (North: 28.40/100 thousand; Northeast: 73.28/100 thousand) is the most frequent, followed by female breast cancer (North: 24.99/100 thousand; Northeast: 52.20/100 thousand) and cervical cancer (North: 20.48/100 thousand; Northeast: 17.59/100 thousand). In the Midwest region, prostate cancer has an estimated risk of 61.60/100 thousand and is the type that most affects the population, followed by female breast cancer (57.28/100 thousand) and colorectal cancer (17.08/100 thousand). 1 2

In relation to the 2023–2025 estimate per year of gynecological cancers specifically, the most frequent are breast cancer 73,610 (30.1%), cervical cancer 17,010 (7.0%), endometrial cancer 7,840 (3.2%) and ovarian cancer 7,310 (3.0%). Estimates for vaginal and vulvar cancers are not available probably given their lower frequency. 2

When comparing with previous estimates (2020–2022) per year of gynecological cancers, there was an increase in the incidence of breast cancer, which was 66,280 (29.7%), of cervical cancer 16,590 (7.4%), endometrial cancer 6,540 (2.9%) and ovarian cancer 6,650 (3.0%). These may be a reflection of the pandemic as, according to Marques et al. (2021), 3 the pandemic period drastically reduced the diagnosis of new cases of cancer in Brazil, possibly given the restrictive measures, including limited consultations in public health services.

Regardless of the difficulties caused by the pandemic, it is important to emphasize that the most frequent gynecological cancers, i.e., breast and cervical cancer, are traceable with consolidated methods such as mammography and oncotic colpocytology/molecular human papillomavirus (HPV) tests, respectively. However, regional socioeconomic disparities result in limitations of access to health services that prevent poor women from being screened, diagnosed and treated.

I emphasize that cervical cancer is a preventable, curable disease with high morbidity and mortality among women in countries without organized prevention programs, such as Brazil. Globally, more than 600,000 new cases appear each year. In August 2020, the World Health Organization (WHO) launched the “Global Strategy to Accelerate the Elimination of Cervical Cancer as a Public Health Problem” based on three pillars: 1) ensure that 90% of girls receive the HPV vaccine by the age of 15; 2) ensure that 70% of women undergo a screening exam with an HPV test by age 35 and another by age 45; and 3) that 90% of women identified with precursor lesions or invasive cancer receive treatment. 4 5 If targets are met, 2 million deaths will be prevented in low- and middle-income countries by 2040. 6 7

Regarding endometrial cancer, the most common gynecological cancer in medium and high socioeconomic development countries, there was an increase in absolute numbers. There is no screening method for endometrial cancer and its risk factors are obesity and a sedentary lifestyle, in addition to diabetes, menarche at an early age, nulliparity, chronic anovulation, late menopause and the use of tamoxifen. Prevention measures such as maintaining weight and physical activity can significantly reduce its risk. Regarding genetic risk, ∼3% of endometrial carcinomas and 5% of cases in women aged under 70 years are predisposed due to hereditary determinants, and these women must be identified, guided and cared for. 8

In relation to ovarian cancer, no single or combined screening strategy has been adequate so far. Family history is the most important risk factor. Other risk factors include reproductive history such as early menarche and late menopause, the use of drugs for the treatment of sterility, hormone therapy after menopause, among others. In addition, there is the hereditary issue, that is, a mutation in the BRCA1 gene confers an estimated risk of 40–50% of developing ovarian cancer up to 70 years of age and the estimate is of 10–20% if the mutation occurs in the BRCA2 gene. 9

Finally, with effective primary and secondary prevention such as lifestyle changes, the HPV vaccine (prevention of induced HPV cancers) and adequate population coverage of screening methods, these rates will decrease significantly. Furthermore, the identification of cancer with a hereditary pattern allows that cancer risk reduction strategies are adopted for patients and their families.
